# A Comparison of Balance and Functional Outcomes After Robotically Assisted Versus Conventional Total Knee Arthroplasty in the Elderly: A Cross-Sectional Study

**DOI:** 10.3390/healthcare13151778

**Published:** 2025-07-23

**Authors:** Gökhan Bayrak, Hakan Zora, Taha Furkan Yağcı, Muhammet Erdi Gürbüz, Gökhan Cansabuncu

**Affiliations:** 1 Department of Physiotherapy and Rehabilitation of the Faculty of Health Sciences, Muş Alparslan University, Muş 49250, Türkiye; 2 Department of Orthopaedics and Traumatology, Kestel State Hospital, Bursa 16450, Türkiye; mdhakanzora@gmail.com; 3 Department of Orthopaedics and Traumatology, Muş State Hospital, Muş 49200, Türkiye; tahafurkanyagci@gmail.com; 4 Department of Orthopaedics and Traumatology, Private Megapoint Hospital, Kahramanmaraş 46100, Türkiye; gurbuzerdi@gmail.com; 5 Department of Orthopaedics and Traumatology, Private Medicana Hospital, Bursa 16110, Türkiye; cansabuncu@gmail.com

**Keywords:** total knee arthroplasty, robotic surgical procedures, physical therapy, balance, surgical outcomes

## Abstract

**Background/Objectives:** Total knee arthroplasty (TKA) is an effective surgical intervention for end stage knee osteoarthritis in elderly patients, with emerging robotically assisted techniques aiming to enhance surgical precision and patient outcomes. This study aimed to compare medium-term balance and functional outcomes between robotically assisted and conventional manual TKA in community-dwelling elderly patients. **Methods:** This cross-sectional study included 50 elderly patients undergoing TKA, who were divided into robotically assisted (n = 25) and conventional manual (n = 25) groups. Demographic and clinical data, balance performance, and functional outcomes were compared at nearly 1.5 years postoperatively. Outcome measures included balance performance assessed by the Berg Balance Scale (BBS), pain via the Visual Analog Scale (VAS), knee function as measured by the Lysholm Knee Scoring Scale, quality of life using the Short Form-12 (SF-12), joint awareness as evaluated by the Forgotten Joint Score-12 (FJS-12), and surgical satisfaction. **Results:** The groups had similar demographic and clinical data regarding age, gender, follow-up duration, surgical time, and anesthesia type (*p* > 0.05). The robotically assisted group demonstrated better balance performance on the BBS (*p* = 0.043) and had a statistically shorter length of hospital stay (1.22 vs. 1.42 days; *p* = 0.005). However, no statistically significant differences were observed in VAS activity pain (*p* = 0.053), Lysholm Knee Scoring Scale (*p* = 0.117), SF-12 physical and mental scores (*p* = 0.174 and *p* = 0.879), FJS-12 (*p* = 0.760), and surgical satisfaction (*p* = 0.218). **Conclusions:** Robotically assisted TKA is associated with advantageous postoperative recovery, particularly in terms of balance performance, showing no clinical difference in other functional outcomes compared to the conventional manual technique. From a physical therapy perspective, these findings emphasize the importance of developing tailored and effective rehabilitation strategies in the medium term for functional recovery in the elderly population.

## 1. Introduction

Total knee arthroplasty (TKA) is performed to relieve pain and enhance physical function in patients with end-stage knee osteoarthritis that limits their ability to perform everyday activities [1]. The conventional manual surgical technique for TKA is an established intervention for patients with knee osteoarthritis and has been the most preferred technique for decades [2,3]. Recently, robotically assisted TKA techniques have emerged, demonstrating improved alignment accuracy and soft tissue balancing compared to conventional manual methods [1,4]. The advent of technology-assisted TKA marks a transformative shift in orthopedic surgery, harnessing the power of computer-assisted navigation and robotic assistance. These advanced methodologies are designed to minimize the variability introduced by surgeons, increasing the precision of implant placement and alignment [5,6].

By improving ligament stabilization and preserving the surrounding soft tissue, innovative robotically assisted techniques may not only present a means to enhance functional outcomes, but may also extend the longevity of implants [7,8]. Robotically assisted evolution in TKA presents a new opportunity for patients seeking greater mobility, better quality of life, and satisfaction [9]. Recent research has shown that incorporating robotically assisted techniques into knee replacement surgery not only simplifies the procedure but also enhances patient outcomes in the short term [3,10]. Additionally, a robotically assisted approach is associated with reduced surgical time and a minimal risk of complications, offering shorter hospital stays, less postoperative pain, and greater function in TKA patients [11,12].

Outcomes such as pain, knee function, and joint alignment have gained significant attention after robotically assisted TKA surgery [8,10]. Functional capacity in elderly TKA patients after surgery may also influence quality of life, joint feeling, and surgical satisfaction, which are essential to postoperative recovery [3,9,13,14]. From the perspective of physical therapy and clinical functional outcomes, although recent studies have indicated early improvements, the clinical advantages of robotically assisted TKA are debated and warrant further exploration [3,8,15]. Nevertheless, there is limited evidence comparing the impact of robotically assisted versus conventional manual TKA on multiple aspects of knee function and balance performance, particularly in the elderly population [2,16]. Therefore, this study aimed to explore balance and functional outcome measures from a physical therapy perspective and assess whether robotically assisted TKA presented better balance performance and functional outcomes than the conventional manual technique.

## 2. Methods

### 2.1. Study Design

This cross-sectional study was conducted in the orthopedics and traumatology clinic of a private tertiary hospital in Türkiye. The Muş Alparslan University Scientific Research and Publication Ethics Committee confirmed the study (protocol code 193289/8-63, approval date 8 May 2025). The study was carried out under the principles of the Declaration of Helsinki. Informed consent was obtained from all patients.

### 2.2. Patients

In 2020, the hospital introduced a robotic arm-assisted TKA system (CORI Surgical System; Smith & Nephew, Memphis, Tennessee). After this introduction, a single experienced surgeon provided all patients with standardized and detailed explanations of both surgical techniques. Patients were then offered both options and allowed to choose their preferred surgical procedure. For the assessments, we identified patients who underwent TKA due to primary gonarthrosis at the hospital between 2021 and 2023 from the hospital’s surgical operation database. We recorded data from community-dwelling elderly patients who underwent TKA using either robotically assisted or conventional manual procedures. The inclusion criteria were admitted patients undergoing TKA due to primary gonarthrosis, being 65+ years of age, and not having any psychiatric or mental disorder that may have affected their responses. The exclusion criteria were revision TKA, previous history of surgery in the operated knee, and superficial and deep infections.

**Figure 1:**
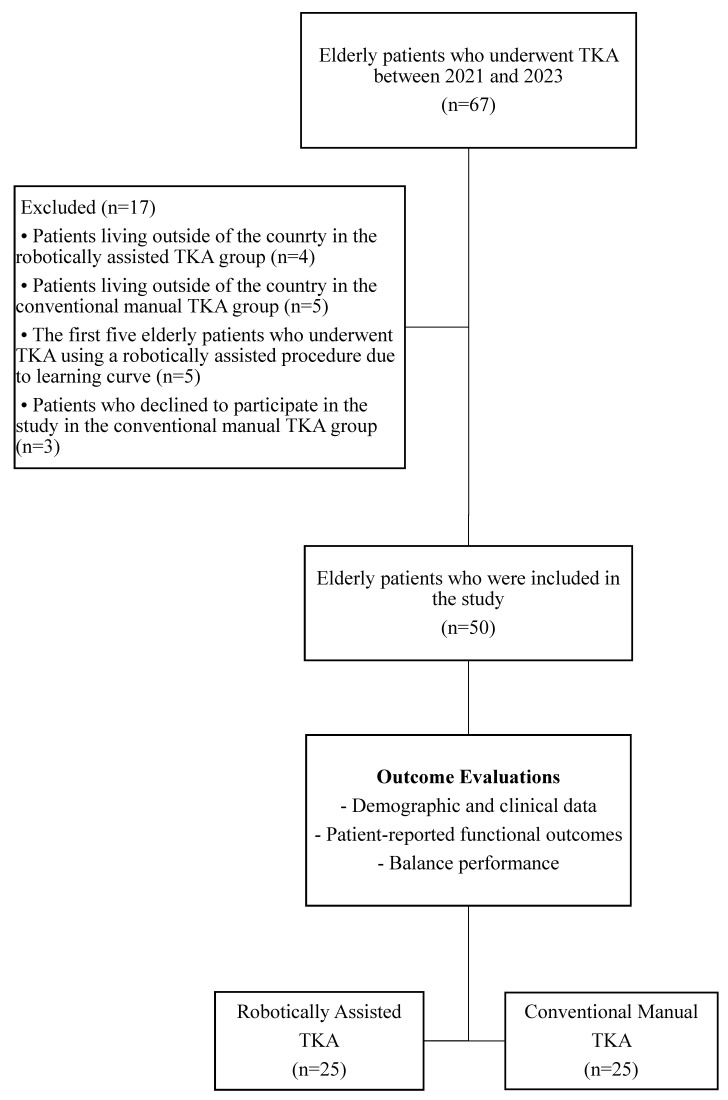
Flow chart of the elderly patients included in the study.

The power ratio of the sample was calculated based on a previous study, and the effect size was determined to be large (d = 0.87) [17]. According to the power analysis, assuming that we could achieve a similar effect size (d = 0.87), *α* level of 0.05, and a power of 0.85, at least 20 patients in the robotically assisted TKA group and at least 20 patients in the conventional manual TKA group were required to complete the study. The eligible 34 elderly robotically assisted and 33 elderly conventional manual TKA patients were called and invited to assessments. Four patients in the robotically assisted and five patients in the conventional manual TKA did not participate due to living outside the country. In the conventional manual TKA group, three patients declined to participate in the study. To eliminate bias associated with the surgeon’s adaptation to the robotic arm-assisted system, the first five elderly patients who underwent robotically assisted TKA were excluded due to the learning curve. A total of 25 robotically assisted and 25 conventional manual TKA patients came to the evaluations, and the outcomes were assessed as outlined in the flow chart (Figure 1).

### 2.3. Surgical Techniques

Our hospital’s orthopedics and traumatology unit is structured into a specialized arthroplasty team comprising a single, well-experienced surgeon with 20 years of experience, a specialist anesthesiologist, assistant physicians, and arthroplasty nurses. This multidisciplinary and specialized surgical team has been assigned solely to arthroplasty patients for many years, contributing to the standardization of a consistent surgical procedure. This specialized surgical team performed all the robotically assisted and conventional manual TKA operations.

#### 2.3.1. Robotically Assisted Technique

All robotically assisted surgeries were performed using the Smith & Nephew CORI surgical system. In all patients, the procedures were conducted under spinal or general anesthesia. An anterior incision was used in all cases, and the joint was accessed via a medial parapatellar arthrotomy. Following the arthrotomy, tibial and femoral pins were placed, along with their corresponding sensors. Tibial and femoral checkpoints were then identified. Anatomical landmarks, including the medial malleolus, lateral malleolus, tibia, and femur, were registered. Through the sensors, data were collected regarding the center of the hip and the flexion–extension range of the knee joint. Subsequently, the femoral and tibial joint surfaces were mapped using a point probe with the smart mapping function, and the data were uploaded into the system. Based on these data, the initial implant planning and positioning were configured using the smart system. Gap balancing in both flexion and extension, under both neutral and varus–valgus stress conditions, was adjusted using the same system. After confirming all parameters, bone cuts were performed using the CORI handpiece burr. The previously planned components were implanted with bone cement. The selected insert was then placed.

#### 2.3.2. Conventional Manual Technique

All surgical procedures were performed under spinal or general anesthesia. Patients were placed in the supine position, and their lower extremities were prepared and draped under sterile conditions. An anterior skin incision was made, and the joint was exposed using a medial parapatellar approach. Femoral and tibial cuts were performed using intramedullary and extramedullary alignment guides, respectively. During bone resections, appropriate valgus angulation was applied, considering the mechanical axis. After achieving soft tissue balance, trial components were inserted to assess the range of motion and stability of the joint. Subsequently, permanent femoral, tibial, and polyethylene components were implanted using bone cement. The surgical site was irrigated with copious saline, and hemostasis was achieved. The procedure was completed by anatomically closing the capsule, subcutaneous tissue, and skin.

### 2.4. Physical Therapy

Postoperatively, both the robotically assisted and conventional manual TKA patients followed the standardized rehabilitation protocol by the same rehabilitation team. However, the exact protocol followed by an individual patient was unique to that patient. During hospitalization, patients started postoperative rehabilitation on the day of surgery. The initial stages of rehabilitation included an ankle pump, active assistive knee flexion and extension range of movement (ROM) exercises, isometric quadriceps setting, and full weight-bearing walking using walkers. After discharge, the patients were started on a two-week home-based physical therapy regimen including active knee ROM exercises, straight leg raise, isometric quadriceps setting, four directional patellar movements, and walking without walkers as tolerated. The stitches were removed on the postoperative 15–18th days. After removal of the stitches, the patients started outpatient physical therapy. The standard outpatient physical therapy included isotonic quadriceps strengthening, calf raises, 5 to 10-cm steps up and down, chair sit-to-stand, high squats, and walking exercises. The standard outpatient physical therapy regimen consisted of 16 sessions for all patients.

### 2.5. Outcome Measures

All outcome measures were prospectively collected at a single time point of assessment, approximately 1.5 years after surgery. The patients’ demographic and clinical data were recorded. The demographic outcomes included age, body mass index (BMI), and operated extremity. The clinical outcomes included anesthesia type, surgical duration, hospital length of stay, use of a stair banister, and the mean follow-up period.

The functional status of the patients was assessed using the patient-reported outcomes. The activity pain levels were evaluated using the Visual Analogue Scale (VAS) [18].

The Lysholm Knee Scoring Scale was used to assess knee function based on eight criteria: pain, instability, locking sensation, swelling, limping, stair climbing, squatting, and need for walking support. Scores range from 0 to 100, where 0 signifies severe dysfunction and 100 indicates optimal function [19].

The Berg Balance Scale (BBS) was used to assess postural control and balance performance, comprising 14 items that focused on static sitting, standing balance, and anticipatory movements during daily activities. Scoring ranges from 0 (unable to perform) to 4 (normal performance), for a total score up to 56 points [20].

The Short Form-12 (SF-12) was used to evaluate patients’ health-related quality of life, consisting of two subheadings: physical and mental health. The SF-12 consists of 12 items related to quality of life, and the total score ranges from 0 to 100, with higher scores indicating a better quality of life [21].

The Forgotten Joint Score-12 (FJS-12) was used to evaluate patients’ awareness of the artificial joint during various daily activities and consists of 12 questions, each using a five-point Likert scale (0–4). The total score is then converted to a scale ranging from 0 to 100, with higher scores indicating a feeling of a more natural or “forgotten” joint [19].

Surgical satisfaction was evaluated using a 100 mm VAS scale, where 0 indicates no satisfaction and 100 indicates complete satisfaction levels.

### 2.6. Statistical Analysis

We performed all statistical analyses for the data using IBM SPSS Statistics version 27.0 (IBM Corp., Armonk, NY, USA). The Shapiro–Wilk test was used to assess the normality of the continuous variables. Descriptive statistics are reported as mean ± standard deviation values for continuous variables and as number (n) and percentage (%) for categorical variables. An independent samples *t*-test was used to explore the means between the two groups. The Pearson chi-square test was used for categorical data analysis. The statistical significance level was set at *p* < 0.05. We conducted a per-protocol analysis, including only patients whose assessments were completed at approximately 1.5 years postoperatively. There were no losses among the patients who agreed to participate in the assessment.

## 3. Results

Demographic, clinical, and functional data for a total of 50 elderly patients were analyzed in the study.

Table 1 presents a comparison of demographic and clinical data between the two groups. The mean age was 71.60 years in the robotically assisted group and 73.28 years in the conventional manual TKA group (*p* = 0.311). The mean follow-up was 16.36 months in the robotically assisted group and 17.60 months in the conventional manual TKA group (*p* = 0.152). The mean surgical duration was 69.16 min in the robotically assisted group and 66.40 min in the conventional manual TKA group (*p* = 0.346). Eighteen patients (72%) received spinal anesthesia and seven patients (28%) received general anesthesia in the robotically assisted group, whereas twenty patients (80%) had spinal anesthesia and five patients (20%) received general anesthesia in the conventional manual group (*p* = 0.508). The mean length of stay was 1.22 days in the robotically assisted group and 1.42 days in the conventional manual TKA group (*p* = 0.005). The BMI (*p* = 0.599), gender distribution (*p* = 0.713), affected extremity (*p* = 0.571), and use of stair banisters during climbing (*p* = 0.089) showed no significant differences between the groups.

Table 2 presents a comparative analysis of balance performance and functional outcomes between the robotically assisted and conventional manual TKA groups. Notably, the robotically assisted group exhibited a better balance performance on the BBS compared to the conventional manual TKA group, with a *p*-value of 0.043. However, the two groups demonstrated comparable results in other functional outcomes, including VAS activity pain levels (*p* = 0.053), Lysholm Knee Scoring Scale (*p* = 0.117), SF-12 physical (*p* = 0.174) and mental scores (*p* = 0.879), FJS-12 (*p* = 0.760), and surgical satisfaction (*p* = 0.218).

The groups had no superficial or deep infections, severe effusions, or deep venous thrombosis. In the robotically assisted group, there was no pin site problem related to the placement of the Schanz guide pin.

## 4. Discussion

This comprehensive study compared the balance performance and functional outcomes following robotically assisted or conventional manual TKA at nearly 1.5 years post-surgery in community-dwelling elderly patients. Notably, the robotically assisted group was associated with better balance performance compared to the conventional manual group. In contrast, the evaluation of functional outcomes—including pain, knee function, quality of life, hospital stay, joint feeling, and surgical satisfaction—revealed comparable results between the two groups.

Achieving and maintaining balance after TKA is a dynamic process, allowing us to perform activities ranging from standing still to engaging in complex physical movements [22]. A recent study comparing robotically assisted versus conventional manual techniques found no specific superiority of one technique over the other in terms of walking balance [2]. Our findings indicated that robotically assisted elderly patients displayed better balance performance compared to those undergoing the conventional manual technique. This is an interesting outcome described for the first time in this study regarding robotically assisted TKA; however, this outcome may not be generalized due to the relatively small sample size. Additionally, the follow-ups for both groups were at nearly 1.5 years, and this outcome could stem from patients’ potential comorbid status, for which we do not have sufficient information. Nevertheless, this is the first study to report balance performance after robotically assisted versus conventional manual TKA in elderly patients.

Robotically assisted TKA adopts the power of functional alignment principles, thereby creating an expectation of improved functional outcomes in patients [1]. For instance, an earlier study noted that patients who underwent robotically assisted TKA experienced less pain and enhanced physical function than those who received conventional manual TKA, as assessed at the 6-month follow-up [17]. Similarly, Choi et al. reported that robotically assisted TKA patients achieved better functional scores than manual TKA at a two-year follow-up [3]. Furthermore, another study reported that robotically assisted TKA patients had good knee functional scores at two years [23] and a 2.5-year follow-up in conventional manual TKA [19]. Supporting these findings, a recent meta-analysis showed that robotically assisted TKA outperformed conventional manual TKA in terms of knee functional outcomes [10]. Conversely, several studies have reported no significant differences in knee function between robotically assisted and manual TKA patients at two-year follow-up [9,15]. In this study, the patients had comparable and adequate knee function and lower knee pain in both robotically assisted and conventional manual TKA at a 1.5-year follow-up. This result may not fully align with earlier findings, which mainly investigate the early phase after surgery. However, the relatively small sample size may have reduced the statistical power to detect a meaningful difference in pain scores. Nevertheless, our findings emphasize the medium-term functional levels and pain status in elderly patients who underwent both types of TKA procedures.

TKA alleviates pain, restores proper joint function, and can significantly enhance a patient’s overall quality of life by effectively addressing osteoarthritis-related issues [24]. Consequently, TKA patients experience enhanced mobility, allowing them to engage in daily activities and enjoy recreation without discomfort, which leads to an improved quality of life [22]. Moreover, a recent study reported that robotically assisted TKA elevates the patient’s quality of life through precise bone cutting and prosthesis positioning [1]. However, earlier research found that the robotically assisted technique improves only two subdomains of the SF-36 quality of life better than the conventional manual TKA at two-year follow-ups [9]. In addition, robotically assisted vs. conventional TKA improved quality of life similarly at an early follow-up [25]. According to normative data, the SF-12 cut-off scores were 50.2 for the mental component in older adults [26] and 26 for the physical component in older patients [27]. Consistent with earlier findings, our results indicated that robotically assisted and conventional manual TKA patients had satisfactory and comparable physical and mental quality of life scores. This similarity may be attributed to factors such as the standardized, early structured physical therapy regimen, the similar nature of long-term functional recovery after joint replacement surgery, and the success of the surgical procedures.

Joint feeling, often characterized as the patients’ subjective assessment of joint stability, mobility, and functionality following surgical intervention, plays a pivotal role in shaping a patient’s quality of life and their capacity to continue daily activities [18]. In recent years, the evaluation of joint feelings after joint arthroplasty has emerged as a significant domain of inquiry, underscoring its relevance as a critical measure in postoperative assessment [19]. Recent studies compared three months [7] and two years of forgotten scores of robotically assisted vs. conventional manual TKA, and indicated that robotically assisted TKA is related to a better joint feeling [3,28] or similar outcomes over two years [15]. Furthermore, patient satisfaction is an important outcome measure and may be influenced by various factors following TKA. Several studies reported that robotically assisted TKA provides superior patient satisfaction at early follow-up [17,29]. However, Song et al. reported no difference between robotically assisted and conventional TKA at over three years of follow-up [30]. Consistent with these findings, our results demonstrated similar joint feelings and patient satisfaction scores in robotically assisted and conventional manual TKA groups at nearly 1.5 years of follow-up.

Length of hospital stay is a frequently evaluated parameter in assessing the efficiency and recovery process. Naziri et al. found that the length of stay was longer, 1.92 days, for patients with conventional TKA, compared to robotically assisted TKA, which was 1.27 days [12]. Likewise, Kayani et al. support this finding and indicated that the robotically assisted technique (3.21 days) was associated with a shorter hospital stay than conventional manual TKA (4.37 days) [11]. In contrast, earlier studies found no significant difference in length of stay between robotically assisted and conventional manual TKA [9,31]. In our study, robotically assisted TKA was associated with earlier hospital discharge (1.22 days), thereby aligning with most of the recent literature. Nevertheless, it is important to note that this difference offers no substantial clinical advantage regarding the type of surgical intervention, postoperative physical therapy perspective, or patient-related functional outcomes.

This study presents crucial insights regarding robotically assisted TKA compared to the conventional manual technique in elderly patients. However, this study has several limitations. First, we do not have the patient’s comorbidity status or preoperative and early postoperative functional outcome data. As the surgical method was determined by patient preference rather than randomization, this may have introduced a selection bias, potentially influencing functional outcomes due to unmeasured patient-related factors. Although our results indicated favorable outcomes in the robotically assisted group, the relatively small sample size may limit the generalizability of these findings. Lastly, our study was restricted to a single-center sample population. Despite these limitations, the findings contribute to a deeper understanding of joint replacement surgery and rehabilitation, emphasizing the clinical implications of robotic assistance in patient outcomes, particularly in terms of superior balance performance for postoperative recovery.

## 5. Conclusions

This study provides a novel perspective into the medium-term outcomes of robotically assisted versus conventional manual TKA in elderly patients. While both surgical techniques yielded comparable results regarding knee function, pain, joint feeling, quality of life, hospital stay, and patient satisfaction, the robotically assisted group demonstrated better balance performance. These findings suggest that robotic assistance is associated with advantageous postoperative recovery, particularly in terms of balance performance. From a physical therapy perspective, the observed balance performance advantage in the robotically assisted TKA could enable the development of more tailored and effective rehabilitation strategies, with potential implications for functional recovery in the medium term in the elderly population. Further large-scale, multicenter, and longitudinal studies, including prospective cohort studies or randomized controlled trials, are needed to expand the understanding of the associations between robotically assisted surgery and postoperative balance performance and functional outcomes.

## Figures and Tables

**Table 1:** Demographic and clinical data of the groups.

Variables	Robotically Assisted TKA (n = 25)		Conventional Manual TKA (n = 25)		*p* ^1^
	Mean ± SD	95% CI	Mean ± SD	95% CI	
Age (year)	71.60 ± 6.51	68.91–74.29	73.28 ± 4.99	71.21–75.34	0.311
Body mass index (kg/m^2^)	25.17 ± 1.34	24.62–25.73	25.37 ± 1.26	24.85–25.89	0.599
Follow-up (months)	16.36 ± 2.78	15.21–17.51	17.60 ± 3.23	16.27–18.93	0.152
Surgical duration (minutes)	69.16 ± 10.04	65.02–73.30	66.40 ± 10.45	62.09–70.71	0.346
Length of stay (days)	1.22 ± 0.11	1.18–1.27	1.42 ± 0.32	1.29–1.56	0.005 *
	n	%	n	%	*p* ^2^ (*χ*^2^)
Gender					
Female	20	80	21	84	0.713 (0.136)
Male	5	20	4	16	
Operated extremity					
Dominant	12	48	14	56	0.571 (0.321)
Non-dominant	13	52	11	44	
Anesthesia type					
Spinal	18	80	20	80	0.508 (0.439)
General	7	20	5	20	
Use of the stair banister					
Yes	9	36	15	60	0.089 (0.885)
No	16	64	10	40	

SD: standard deviation; 95% CI: 95% confidence interval for means; kg: kilogram; m: meter; *p* ^1^: independent samples *t*-test significance value; *p* ^2^: Pearson Chi-square test statistical significance value; *χ*^2^: Pearson Chi-square test statistics. *: statistical significance.

**Table 2:** Comparison of balance performance and functional outcomes between the groups.

Variables	Robotically Assisted TKA (n = 25)		Conventional Manual TKA (n = 25)		*p*
	Mean ± SD	95% CI	Mean ± SD	95% CI	
Berg Balance Scale score	37.28 ± 5.08	35.18–39.38	33.84 ± 6.56	31.13–36.55	0.043 *
VAS activity pain (mm)	8.18 ± 5.98	5.71–10.64	11.44 ± 5.64	9.11–13.77	0.053
Lysholm Knee Scoring Scale	77.48 ± 5.78	75.09–79.87	74.88 ± 5.74	72.51–77.25	0.117
SF-12 physical score	39.72 ± 4.80	37.74–41.70	37.39 ± 6.94	34.52–40.25	0.174
SF-12 mental score	51.58 ± 5.39	49.35–53.80	51.33 ± 5.86	48.91–53.75	0.879
Forgotten Joint Score-12	49.37 ± 9.20	45.57–53.17	50.17 ± 9.16	46.38–53.95	0.760
Surgical satisfaction (mm)	88.72 ± 7.09	85.79–91.65	86.16 ± 7.41	83.10–89.22	0.218

SD: standard deviation; 95% CI: 95% confidence interval for means; mm: millimeter; *p*: independent samples *t*-test significance value; *: statistical significance.

## Data Availability

Due to privacy and ethical restrictions, the data are not publicly available.
